# When ISG15 is involved in inflammation

**DOI:** 10.3389/fimmu.2026.1775143

**Published:** 2026-04-14

**Authors:** Xueting Feng, Yingli Qiao, Shufang Cui

**Affiliations:** 1 State Key Laboratory of Natural Medicines, School of Life Science and Technology, China Pharmaceutical University, Nanjing, China; 2 Department of Laboratory Medicine, Henan Provincial People’s Hospital, Fuwai Central China Cardiovascular Hospital, Henan, Zhengzhou, China

**Keywords:** ISG15, ISGylation, skin inflammation, cardiovascular inflammation, neuroinflammation

## Abstract

Interferon-stimulated gene 15 (ISG15), a key ubiquitin-like modifying molecule, plays an important role in regulating inflammatory responses. This review summarizes the functions of ISG15 in different inflammatory diseases. On the one hand, ISG15 precisely regulates the activity of signaling proteins through its intracellular modification function, thereby affecting the type I interferon signaling pathway; on the other hand, free extracellular ISG15 can act as a cytokine, activating immune cells and exacerbating inflammatory responses. We further explored the specific mechanisms of ISG15 in skin inflammation, cardiovascular inflammation, neuroinflammation, and other types of inflammation and analyzed the limitations of current studies. Finally, this study highlights the potential value of targeting the ISG15 pathway as a new strategy for the treatment of inflammatory diseases.

## 1 Introduction

Interferon-stimulated genes (IFN-stimulated genes, ISGs) are genes with lengths of more than 500 bp that are stimulated by type I interferons (I-IFNs). Among these ISGs, ISG15 was first discovered and is now widely studied (1). Lengyel P and coworkers first reported a protein in interferon-treated Ehrlich ascites tumor cells in 1979, whereas they did not investigate the function of this unnamed protein (2). Later, Arthur L. Haas and colleagues identified the astonishing similarity between this protein and ubiquitin, thus naming it ubiquitin cross-reactive protein (UCRP) (3). It was not until 1987 that the name ISG15 first appeared, as it is a member of the ISG family that encodes a 15 kDa protein (4). I-IFNs are produced and secreted when cells sense external pathogen-associated molecular patterns (PAMPs) or internal damage-associated molecular patterns (DAMPs) via the host germline-encoded pattern recognition receptor (PRR). Extracellular I-IFNs interact with the interferon receptor (IFNAR) on the cell membrane and activate the downstream Janus kinase-signal transducer and activator of transcription (JAK-STAT) signaling pathway. Phosphorylated STAT1 and STAT2 interact with interferon regulatory factor 9 (IRF9), leading to the formation of a ternary complex called interferon-stimulated gene factor 3 (ISGF3). ISGF3 enters the nucleus and recognizes the interferon-sensitive response element (ISRE) promoter of ISG15, which can induce its transcription and translation (5). In addition to I-IFNs, III-IFNs can also induce the production of ISG15 (6).

**Figure 1:**
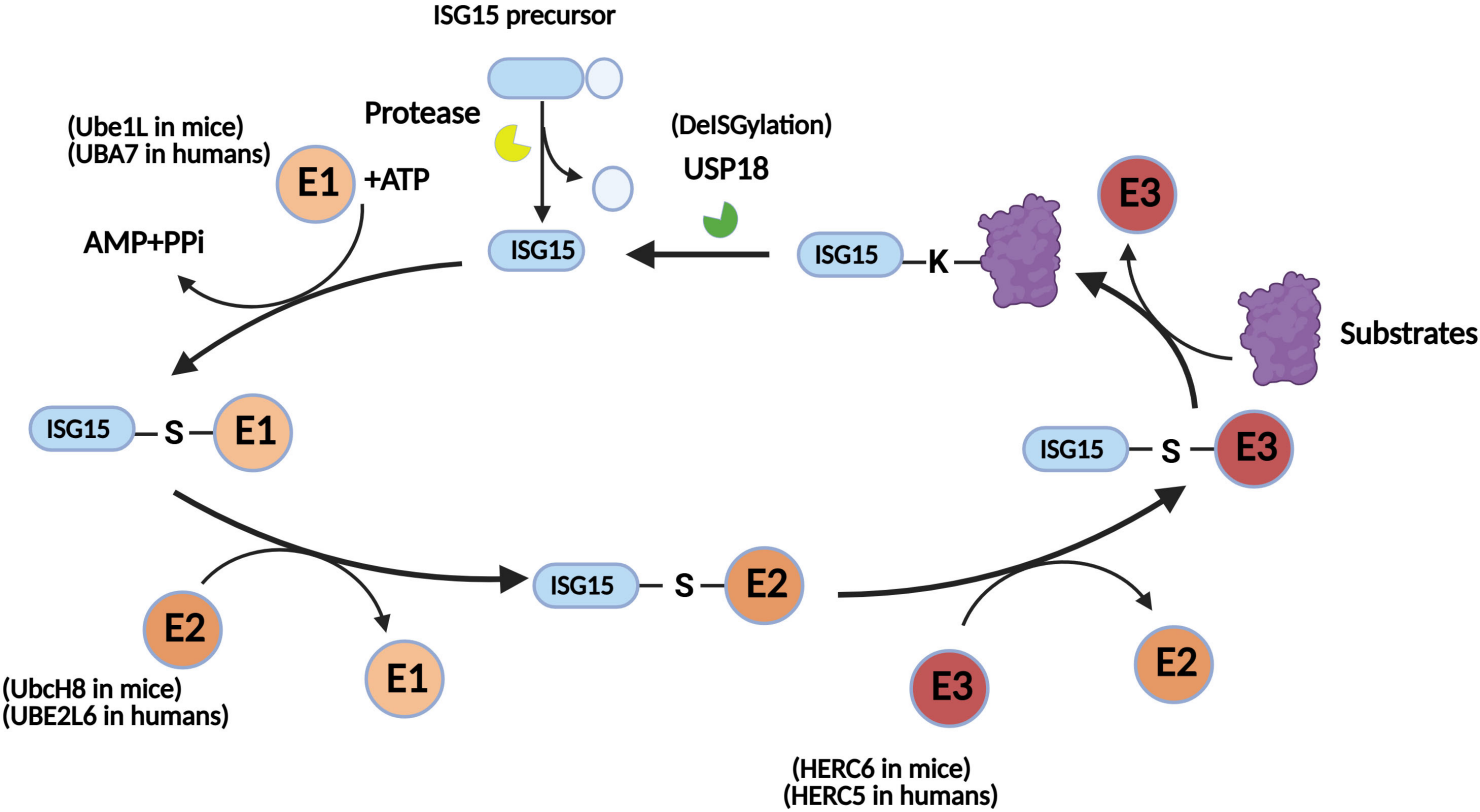
The process of ISGylation. The ISG15 precursor is processed by a protease to form mature ISG15. Mature ISG15 consumes ATP and is connected to E1 through high-energy thioester bonds. ISG15 is transferred from E1 to E2, then to E3, and finally to a lysine residue of the target protein. This process can be reversed by USP18.

ISG15 has two forms *in vivo*: the free form and the conjugated form. The conjugated form of ISG15 refers to its binding of substrate proteins via covalent bonds within cells, whereas free ISG15 is distributed both inside and outside the cell. ISG15 conjugation also known as ISGylation, which is a reversible posttranslational modification similar to ubiquitination. Classic ISGylation is a three-step enzymatic cascade that requires the participation of E1-activating enzymes (Ube1L in mice or UBA7 in humans), E2-conjugating enzymes (UbcH8 in mice or UBE2L6 in humans), and E3 ligases (HERC6 in mice or HERC5 in humans) (7). In this process, E1 consumes energy generated by ATP and forms a high-energy thioester bond with glycine residues at the carboxyl terminus of ISG15. The activated ISG15 is subsequently transferred to a cysteine residue on E2, and then ISG15 is transferred to E3 by transesterification. E3 specifically recognizes and transfers ISG15 to the lysine residue of target proteins to regulate their functions (8) (**Figure 1**). This progress can be reversed by ubiquitin-specific peptidase 18 (USP18), which negatively regulates I-IFNs and III-IFNs (9, 10). Notably, this negative regulation is independent of its deISGylation activity. USP18 competes with I-IFNs for binding to interferon alpha and beta receptor subunit 2 (IFNAR2), thereby inhibiting the activation of the downstream JAK-STAT signaling pathway (11). Extracellular free ISG15 exerts effects similar to those of cytokines by binding to receptors on the membrane, such as lymphocyte function-associated antigen 1 (LFA-1) receptors (12, 13).

As ISG15 can be induced mainly by I-IFNs, sometimes by III-IFNs (14) or genotoxic stressors (15), thus under normal physiological conditions, the expression level of ISG15 protein is extremely low and rarely detected (1). Under pathological circumstances such as viral and bacterial infections, stress, tumors and inflammatory diseases, both immune cells and non-immune somatic cells can be stimulated to produce ISG15. For example, ISG15 can be produced by mycobacterium-treated leukocytes-granulocytes (16), plasma cells of patients with active systemic lupus erythematosus (17), lymphocytes of goats infected with Peste-Des-Petits-Ruminants Virus (18), and epithelial cells in the C. trachomatis-infected mouse (19). In addition, tumor cells can also secrete ISG15 (20).

Considering that the induction of ISG15 depends on interferon pathways, people naturally think of its antiviral role (21, 22), which has also been the focus of previous research. In recent years, it has been reported that ISG15 also functions in other pathologic circumstances, such as tumors, neurodegeneration and inflammation (23–26). In this study, we elaborate on the role of this protein in inflammation.

## 2 ISG15 and inflammation

**Figure 2:**
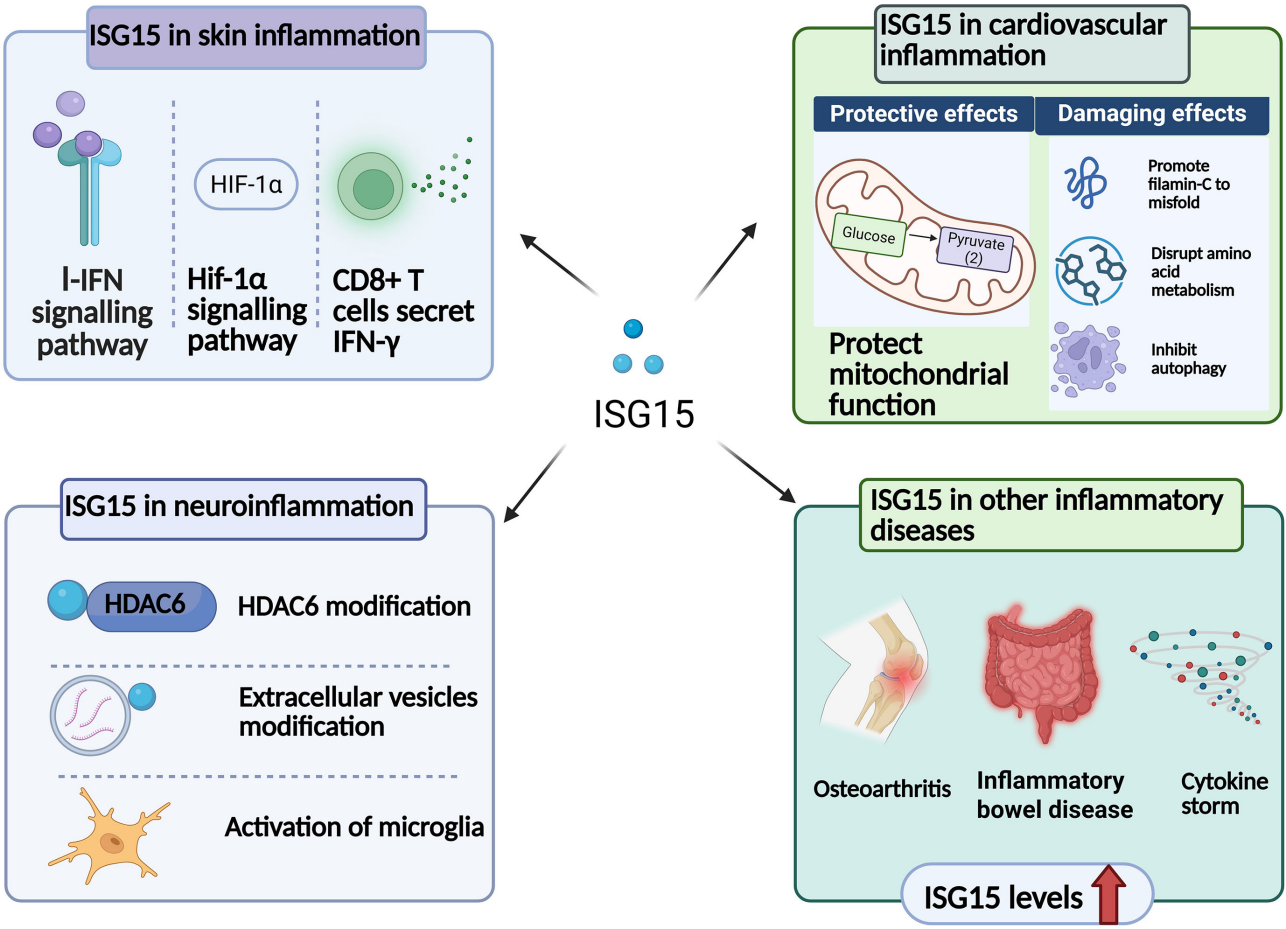
The function and regulatory mechanism of ISG15 in different dieases. ISG15 is involved in the pathogenesis of skin inflammation through its association with the I-IFN signaling pathway, HIF-1α signaling, and IFN-γ secreted by CD8^+^ T cells. In cardiovascular inflammation, ISG15 has both protective and damaging effects. In neuroinflammation, ISG15 can modify HDAC6 and extracellular vesicles and activate microglial. ISG15 is also associated with osteoarthritis, inflammatory bowel disease and cytokine storm.

Many studies have shown that ISG15 is closely related to different types of inflammation, such as skin inflammation, cardiovascular inflammation and neuroinflammation. For example, patients with ISG15 deficiency may present with inflammatory skin ulcers and lesions (27). The function and regulatory mechanism of ISG15 are diverse in different inflammatory diseases (**Figure 2**), largely depending on the immune microenvironment. Among the complex molecular mechanisms by which ISG15 regulates inflammation, the implications of ISG15 in autophagy are investigated extensively and deserves more attention. In some microbe-induced inflammatory diseases such as *T. gondii* infection, ISG15 can promote autophagy by binding to key autophagy-related proteins p62 and histone deacetylase 6 (HDAC6), to form ISG15 aggregates or aggresomes. The p62 in these ISG15 aggregates or aggresomes then recruits autophagosome-bound LC3, which is beneficial to the formation of autophagosomes. HDAC6 can mediate the fusion of lysosomes containing ISG15 aggregates via its deacetylase activity, thereby promoting the clearance of substrate proteins bound to ISG15 (28, 29). In addition, in the animal model infected with *Listeria*, ISG15 can also bind to the key regulatory molecules of autophagy, mammalian target of rapamycin (mTOR) and WD repeat domain phosphoinositide interacting 2 (WIPI2), leading to activation of the autophagy pathway (30). However, ISG15 can inhibit autophagy in some non-infectious inflammation, such as in Alzheimer’s disease (AD), where elevated ISG15 binds to HDAC6 and inhibits its activity. This leads to excessive acetylation of downstream cortactin and hindering the fusion of autophagosomes-lysosomes, ultimately obstructing autophagic flow and resulting in the accumulation of toxic protein tau (31). ISG15 can exert positive or negative effects on the autophagy process by modifying different proteins in different inflammatory diseases, and we will discussed about these detailedly below (31–33).

### 2.1 ISG15 and skin inflammation

It has been reported that functional defects in the ISG15 gene in humans can lead to extensive or recurrent skin ulcers or even necrotic lesions (34–39). These skin lesions are phenotypes of the autoinflammatory process caused by overactivation of I-IFN signaling. It is believed that ISG15 can stabilize USP18, which negatively regulates the I-IFN signaling pathway. Thus, patients with genetic ISG15 deficiency have lower USP18 and higher I-IFN levels than healthy individuals do (40). In other words, the genetic absence of ISG15 results in a failure to inhibit I-IFN signaling, leading to sustained inflammation and damage to skin cells. Studies on skin cells from ISG15-deficient models or patients have revealed a “high inflammation” phenotype. In these cells, the expression of genes and proteins involved in skin structure, cell adhesion, the basement membrane and collagen is abnormal (35–41). ISG15^-/-^ fibroblasts have a reduced ability to migrate and control reactive oxygen species (ROS) (35). In 3D epidermal models, the genetic absence of ISG15 leads to poor connectivity, a lower density of bridge particles, and a looser epidermal structure (35, 41–43). Two compound heterozygous variants (c.285del and c.299_312del, NM_005101.4 GRCh37(hg19)) in the ISG15 gene lead to a complete loss of the ISG15 protein. This deficiency results in ulcerative skin lesions (27). In contrast, an abnormal increase in ISG15 has been reported to be closely related to other skin inflammatory diseases (44–50). In some studies of autoimmune dermatomyositis (DM), the ISG15 expression was upregulated in the skin biopsy or muscle tissue. Notably, the levels of ISG15 and ISG15 conjugated-proteins are elevated in cultured human skeletal muscle stimulated by I-IFN, suggesting that ISGylation may be involved in the pathological progression of dermatomyositis (51). In patients with anti-melanoma differentiation-associated gene 5-positive dermatomyositis (MDA5^+^DM), a higher proportion of CD8^+^ T cells expressing ISG15 in the skin is linked to poorer prognosis, which is also due to the activation of the I-IFN signaling pathway (52). The number of monocytes that overexpress ISG15 is significantly increased in the skin of patients with Behçet’s disease (BD), which is a chronic systemic vasculitis (53).

**Figure 3:**
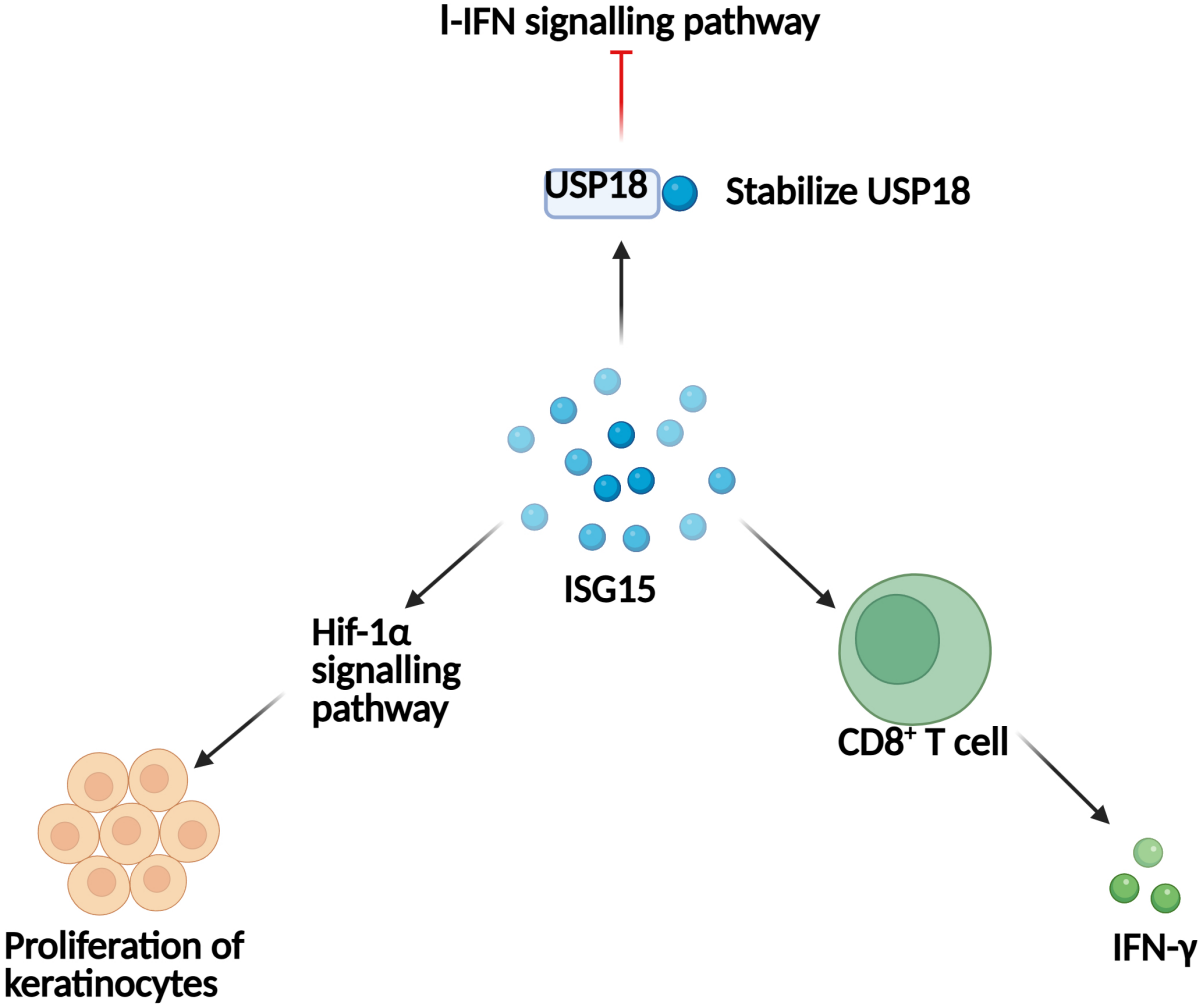
The mechanism by which ISG15 regulates skin inflammation. ISG15 stabilizes USP18, promotes the secretion of IFN-γ by CD8^+^ T cells, and regulates the Hif-1α signaling pathway, thereby affecting skin inflammation.

On the basis of current evidence, several possible mechanisms can be proposed to explain the relationship between ISG15 and skin inflammation (**Figure 3**). First, ISG15 stabilizes USP18 under normal conditions, thereby inhibiting the I-IFN signaling pathway and preventing overactivation of the I-IFN response (40). The absence or dysfunction of ISG15 can cause instability of USP18, which in turn fails to suppress I-IFN signaling properly, leading to its persistent activation and ultimately chronic autoinflammation. The persistent high level of IFN signaling caused by ISG15 deficiency can trigger inflammatory cell activation, decrease collagen and adhesion molecules that maintain skin structure (35), which can then damage keratinocytes or basal cells and ultimately cause destruction of skin tissue. Second, ROS may participate regulating the pathological process of ISG15-related skin inflammation. On one hand, ISG15 deficiency results in higher ROS levels and a reduced function of mitochondria, which subsequently triggers an increase in cell apoptosis/pyroptosis (54). On the other hand, oxidative stress can induce hypermethylation of the USP18 promoter region, thus increase ISG15 expression in keratinocytes and melanocytes and ultimately exacerbates vitiligo (55). Mechanically, the increased ISG15 acts as cytokine by binding to LFA-1 receptors on the surface of CD8^+^ T cells, thus inducing the polarization of CD8^+^ T cells towards type I T helper (Th1) cell subsets and enhancing the production of pathogenic cytokine IFN-γ (55, 56). Noteworthy, the increased ISG15 functioned as “double-edged sword” in some skin inflammation. For example, IL-29 produced by Th17 cells can promote the expression of ISG15 in epithelial cells, thereby strengthening the antiviral ability of psoriasis skin (37). However, the presence of ISG15 can promote the proliferation of keratinocytes and the expression of cell cycle-related protein-cyclin D1 through the hypoxia inducible factor-1α (Hif-1α) signaling pathway, thereby exacerbating psoriasis (42).

Although current evidence supports the important role of ISG15 in skin inflammation, many unanswered questions remain. Most academic reports focus on extreme cases of ISG15 deficiency or functional impairment, but direct research on the role of ISG15 in skin inflammatory diseases, such as vitiligo, atopic dermatitis, psoriasis and melanoma, is still relatively limited. The role of ISG15 in different types of skin cells may have significant heterogeneity and has not yet been fully elucidated. The covalent modification targets of ISG15 in skin tissue are not yet clear, which limits the clarification of the precise mechanism involved. In addition, there is currently no clear clinical strategy for how to intervene in skin inflammation by regulating ISG15 or its downstream pathways.

### 2.2 ISG15 and cardiovascular inflammation

Recent studies have shown that aberrant expression of ISG15 in several cardiovascular inflammatory processes, such as atherosclerosis, myocardial infarction, myocarditis and myocardial remodeling (33, 57–62).

ISG15 and ISGylation are increased in myocardial tissue under pressure loading and in vascular remodeling models. Metabolic analysis further demonstrated that in a transverse aortic constriction model, the hearts of ISG15^-/-^ mice differed from those of wild-type mice in terms of amino acid metabolism, such as D-glutamine and D-glutamate metabolism, β-alanine metabolism, glutathione metabolism, and nitrogen metabolism (33). In the analysis of 22 immune cell types within atherosclerotic tissue, both memory B cells and resting dendritic cells were positively correlated with ISG15 expression levels (57). The IFN pathway is activated in cardiomyocytes via NF-κB, promoting ISG15 expression and driving myocarditis. This association between NF-κB and ISG15 has been further validated in a murine myocarditis model (61). ISG15 antagonizes the ubiquitin proteasome pathway and works together with STAT1, which inhibits autophagy, leading to myocardial cell death in myocardial infarction (59). One key target of ISG15 is the cardiomyocyte myofibrillar protein filamin-C, which functions by maintaining the myocardial structure. ISGylation alters the folding and conformation of filamin-C, thus promoting the accumulation of misfolded proteins in cardiomyocytes. Genetic ISG15 deficiency decreases the accumulation of misfolded proteins, and autophagy in cardiomyocytes is promoted to help clear damaged proteins (33, 57–63). ISG15 and ISGylation can increase oxidative stress and induce the expression of inflammatory markers, thus affecting vascular remodeling and contributing to endothelial dysfunction (64).

Furthermore, in the Coxsackie B3 virus (CVB3)-infected cardiomyopathy model, the ISG15 system was strongly activated. ISG15^-/-^ mice infected with CVB3 develop functional defects such as myocardial atrophy and decreased cardiac output (60). ISG15 covalently modifies two glycolytic enzymes, hexokinase 2 (HK2) and phosphofructokinase muscle form (PFK1), thus inhibiting the activity of these two enzymes and limiting interferon-induced glycolysis. Furthermore, ISGylation of HK2 and PFK1 can simultaneously protect mitochondrial function and oxidative phosphorylation ability, allowing the myocardium to maintain ATP production even under nutrient limitation or metabolic stress (22).

**Figure 4:**
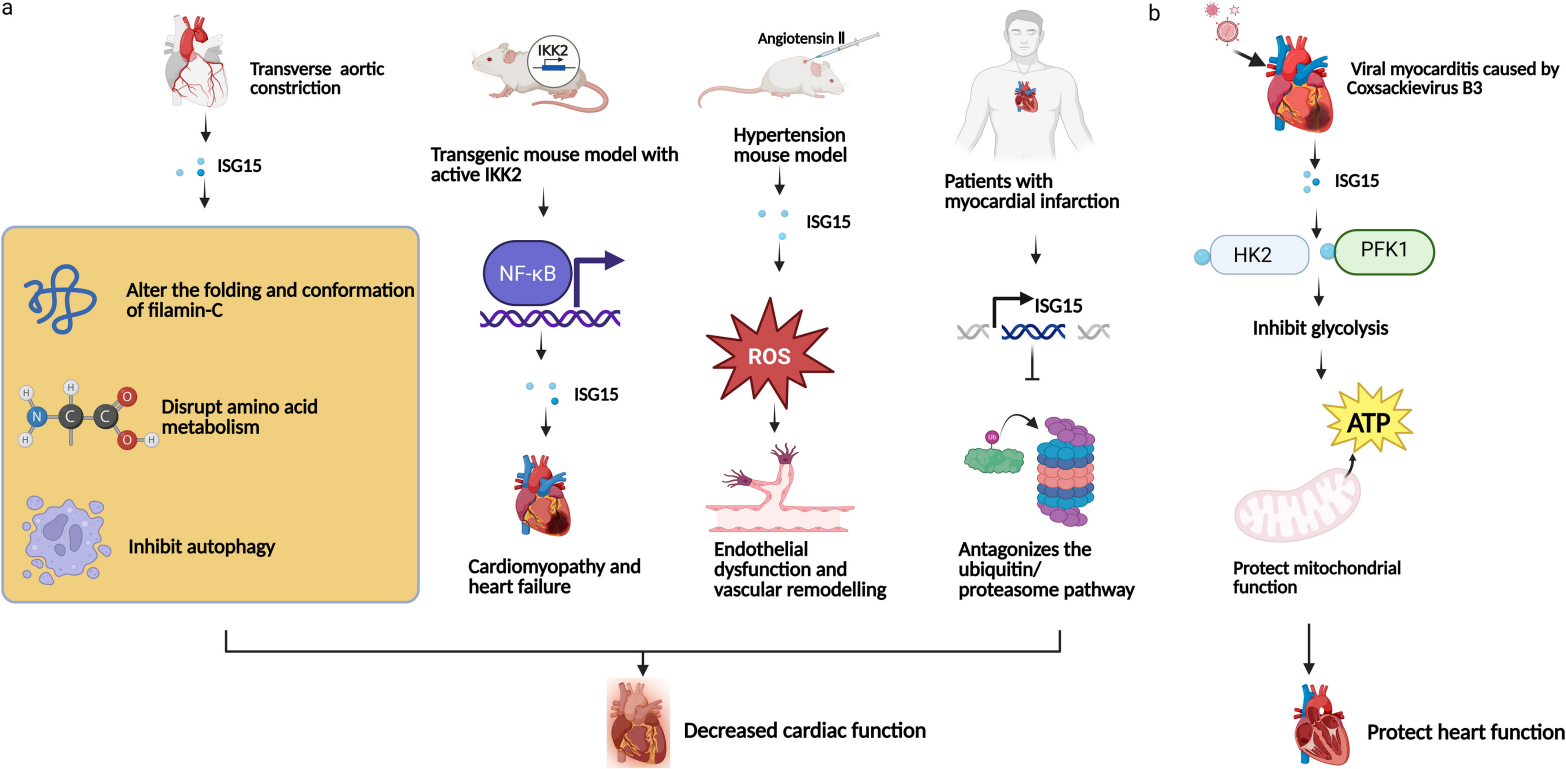
ISG15 plays dual roles in different cardiovascular inflammation models. **(a)** The detrimental function of ISG15. The level of ISG15 increases under conditions of transverse aortic constriction, thus promoteing filamin-C misfolding, disrupting amino acid metabolism and inhibiting cellular autophagy. In transgenic mouse model, activation of the NF-κB signaling pathway leads to increased expression of ISG15. In hypertensive mouse model, ISG15 increases the reactive oxygen species (ROS) levels, leading to endothelial damage and vascular remodeling. ISG15 inhibits the ubiquitin proteasome pathway in patients with myocardial infraction. **(b)** The protective function of ISG15. The ISG15 expression is upregulated during viral myocarditis, leading to crippled glycolysis and enhanced mitochondrial function by covalently modifying HK2 and PFK1.

The above studies revealed that ISG15 plays diverse roles in cardiovascular disease, which is dependent on pathological circumstances (**Figure 4**). ISG15 expression is increased in models of stress overload, high angiotensin II levels and heart failure. Increased ISG15 contributes to filamin-C misfolding or NF-κB activation, disrupts amino acid metabolism or inhibits autophagy in heart muscle cells, which accelerates pathological remodeling and functional decline. In contrast, during viral myocarditis or metabolic stress, ISG15 can reduce heart dysfunction by regulating metabolism and preventing severe nutrient or oxygen shortages. These findings suggest that clinical interventions, such as regulating ISG15 expression or ISGylation activity, may offer a new approach to improve cardiovascular diseases.

### 2.3 ISG15 and neuroinflammation

Recent progresses have revealed that ISG15 functioned in central nervous system (CNS) inflammation through both conjugated and non-covalent forms. On the one hand, it regulates intracellular autophagy and affects nucleic acid-sensing pathways through ISGylation. On the other hand, free ISG15 can act as an extracellular signal on microglia, promoting or shaping neuroinflammatory responses. The upregulation of ISG15 has been observed in various neuroinflammatory or neurodegenerative conditions, such as Aicardi-Goutières syndrome (AGS), neuroinflammation caused by viral infection, traumatic brain injury (TBI), Alzheimer’s disease (AD), and multiple sclerosis (MS), suggesting that ISG15 functions in neuroinflammation (65–70).

ISG15 mRNA is upregulated in Aicardi-Goutières syndrome (AGS), which is a rare genetic neurological disease characterized by elevated levels of I-IFN (65). In the brains of IFN-deficient mice infected with Theiler’s murine encephalomyelitis virus (TMEV), the expression levels of ISG15 are greater than those in the brains of wild-type mice, suggesting that ISG15 may be involved in preventing the disease progression of encephalitis caused by TMEV (66). The ISG15 levels of lymphocytes increase in the brains stimulated with human immunodeficiency virus (HIV-1), largely due to CD4^+^ cytotoxic T lymphocytes express more LFA-1 and Very Late Antigen-4 (VLA-4) after infection, thereby facilitating the adhesion of lymphocytes to vascular endothelial cells and lymphocytes crossing the blood-brain barrier (69). In traumatic brain injury (TBI), an increase in ISG15 was also observed (70). Research has revealed that the cGAS-STING pathway, which is upstream of ISG15, contributes to the development of multiple neuroinflammatory diseases, including Alzheimer’s disease, multiple sclerosis, and Parkinson’s disease (71–73). Increased STING expression was observed in AD model mice. Further evidence suggests that amyloid-β (Aβ) and tau protein accumulation in AD leads to cytoplasmic DNA accumulation, activating the cGAS-STING pathway and increasing the levels of downstream inflammatory factors, thereby aggravating the disease (71). Notably, STING inhibitors can effectively mitigate AD symptoms (71). One of the characteristics of Parkinson’s disease is the abnormal aggregation of α-synuclein (αSyn) (74). Increased STING levels are observed both in microglia treated with αSyn preformed fibrils and in Parkinson’s disease patients with αSyn accumulation (72). Similarly, activation of the SING pathway can also be observed in endothelial cells and nerve cells in MS (73). In turn, ISG15 can modify STING through ISGylation, which stabilizes STING by preventing its degradation and further influences neuroinflammation via nucleic acid-sensing pathways (75). In an Alzheimer’s disease (AD) model, the accumulation of the microtubule-associated protein tau (MAPT) contributes to elevated ISG15 levels. The increased ISG15 covalently interacts with histone deacetylase 6 (HDAC6), which is involved in regulating the autophagy-lysosome pathway. The ISGylation of HDAC6 and accompanying reduction in its acetylation inhibits HDAC6 activity, leading to obstruction of autophagosome-lysosome fusion by disrupting cortactin and F-actin recruitment to lysosomes. The impairment of autophagy further prevents MAPT degradation, forming a vicious cycle and ultimately resulting in various phenotypes, such as blockade of autophagy flow, neuronal damage and cognitive impairment (31). In a multiple sclerosis (MS) model, demyelination led to increased ISGylation in neurons (68). We previously reported that cGAS colocalized with ISG15 in the demyelination spinal cord in an MS mouse model, suggesting that the ISGylation of cGAS may participate in MS progression (76). The ISG15 dimer secreted by neurons can bind to and activate CD11b on the surface of microglia, resulting in the upregulation of proinflammatory factors such as iNOS. INOS can increase the level of NO in the cytoplasm, causing ISG15 nitrosylation, which can, in turn, inhibit the formation of ISG15 dimers and promote ISGylation. Moreover, extracellular vesicles with ISGylated surface proteins contain more proinflammatory and neurotoxic miRNAs, while the levels of anti-inflammatory and neuroprotective miRNAs are lower in these vesicles than in ISG15-knockdown neurons (68).

**Figure 5:**
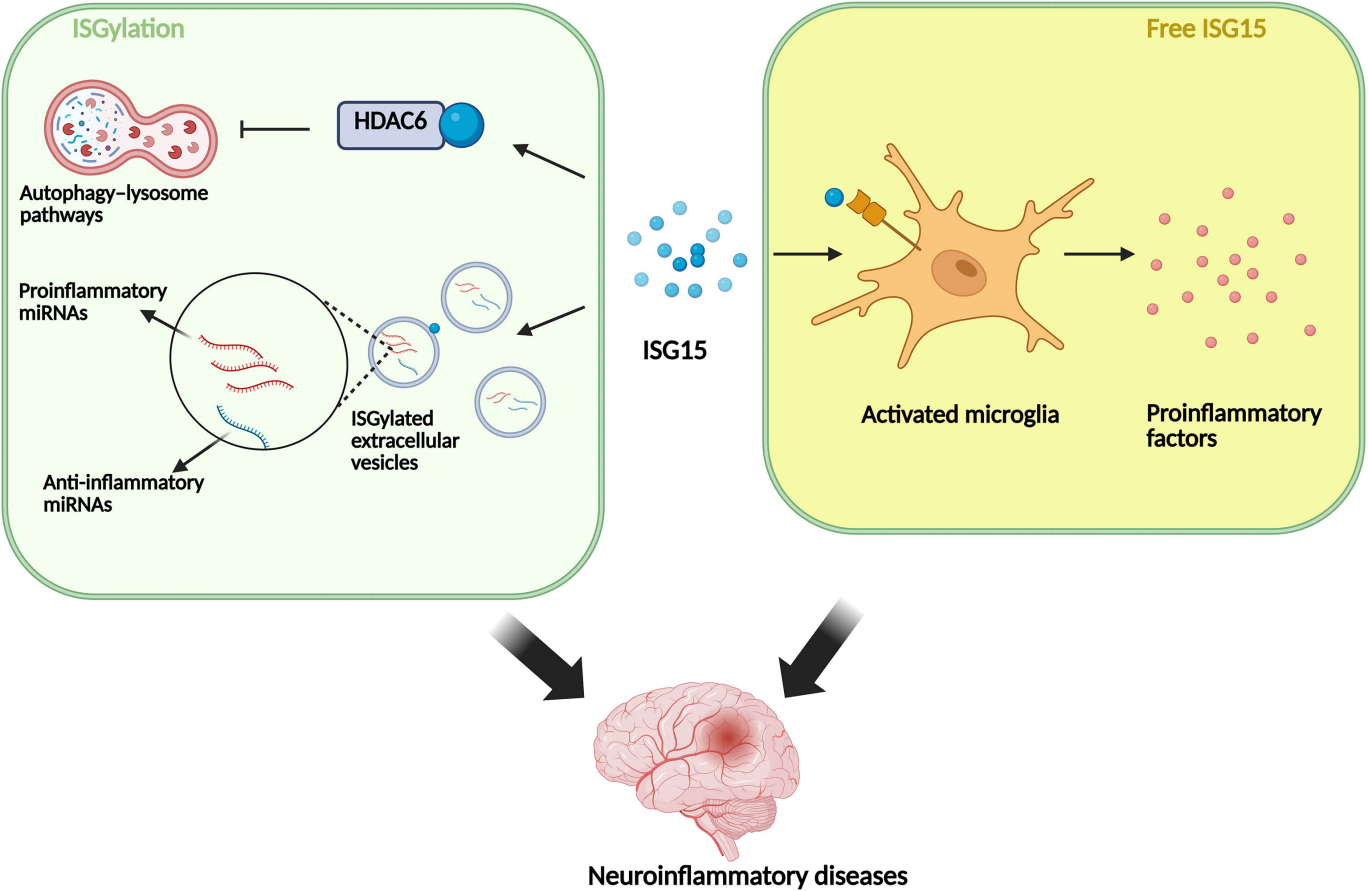
Role of ISG15 in neuroinflammation. ISGylation of HDAC6 can inhibit the autophagosomal pathway. ISGylation of proteins on the neuronal extracellular vesicles can increase the levels of proinflammatory and neurotoxic miRNAs within the vesicles while reducing the levels of anti-inflammatory and neuroprotective miRNAs. Free ISG15 can activate microglia and secrete proinflammatory cytokines. Both forms of action of ISG15 can exacerbate neuroinflammation.

On the basis of the above evidence (see **Figure 5**), ISG15 in neurons may inhibit the activity of HDAC6, leading to the obstruction of autophagy-lysosome pathways and resulting in increased cytotoxicity. ISG15 may also regulate EVs released by neurons, which can regulate the inflammatory state of microglia. Secreted free ISG15 can act on other immune cells and regulate their activation status. Moreover, ISGylation may stabilize or interfere with the degradation of certain inflammatory regulatory proteins, thereby enhancing inflammatory signals. The central node characteristics of ISG15 in the immune nervous system make it a potential target for connecting peripheral immunity, neuronal-glial interactions, and balancing inflammatory responses. However, the identified targets of ISG15 are relatively limited, and identifying and interfering with the key targets of ISG15 may constitute a new strategy for treating neuroinflammatory diseases.

### 2.4 ISG15 and other inflammatory diseases

In addition to its role in neuroinflammation, skin inflammation, and cardiovascular inflammation, recent studies revealed that ISG15 may function in several other inflammatory diseases.

Based on multiomics screening, ISG15 has been identified as a biomarker for inflammatory bowel disease (IBD), which is associated with immune infiltration (36). In addition, exposure to the estrogen-like mycotoxin zearalenone (ZEN) can upregulate the STAT2/STAT6 and ISG15 proteins in the colons of rats, suggesting that mucosal inflammation associated with the IFN/STAT-ISG15 axis may increase the risk of IBD (77). Longitudinal transcriptome monitoring revealed that ISG15⁺ HLADR^hi^ monocytes repeatedly appear in the blood of rheumatoid arthritis (RA) patients with periodontal disease. Considering that RA patients are more likely to suffer from periodontal disease, it can be inferred that the presence of ISG15⁺ HLADR^hi^ monocytes is related to an increased incidence of periodontal disease in RA patients (78). ISG15 can also block PINK1/Parkin-mediated mitochondrial autophagy, which promote cartilage degradation and exacerbate osteoarthritis (79). Moreover, the upregulation of ISG15 during fetal development can induce an endogenous cytokine storm and fetal liver hematopoietic failure, leading to embryonic death (80). Chronic inflammation is associated with tumorigenesis, and the upregulation of ISG15 through the IFN pathway is believed to affect tumor development and treatment response. ISG15 can amplify inflammation and help tumor cells adapt to inflammation and replication pressure, making it an important regulatory node and potential therapeutic target for inflammation-related tumors (81, 82).

## 3 Summary and prospective

In summary, ISG15 plays diverse roles in different inflammatory diseases through either the ISGylation of key targets or its function as a cytokine (**Table 1**). Despite progress, the role of ISG15 in different pathological states remain unclear, especially the dynamic balance between proinflammatory and anti-inflammatory functions. What’s more, the cellular sources and release mechanisms of extracellular ISG15 *in vivo*, particularly in non-immune cells and tissue-specific microenvironments, are not well defined. In addition, most of the current research relies on animal models, and the mechanism of ISG15 in human inflammatory diseases still needs to be further verified.

**Table 1:** The form of ISG15 in different inflammations.

Skin inflammation	ISG15 form	ISG15 function	Research model	Refs
Systemic sclerosis	N/A	The ISG15 levels increase in systemic sclerosis	Cells from systemic sclerosis patients	(44)
Psoriasis	N/A	The ISG15 levels increase in psoriasis	Cells from psoriasis patients	(45, 46)
	N/A	Promote the proliferation of keratinocyte through Hif- 1α pathway	Mice and cell models of psoriasis	(42)
Systemic lupus erythematosus	N/A	The ISG15 levels increase in mice models with skin lesions	Skin lesions of oxyguanine glycosylase 1^-/-^ (*OGG1^-/-^*) mice	(47)
Chronic autoimmune urticaria	N/A	The ISG15 levels increase in urticaria mice models	Chronic autoimmune urticaria patients’ skin tissues and mice models	(49)
Dermatomyositis	Conjugated	Increase the I-IFN level	Muscle biopsy specimens from dermatomyositis patients	(51)
Behçet disease	N/A	The ISG15 levels increase in Behçet patients	Skin samples from Behçet disease patients	(53)
Vitiligo	Free	Promote IFN-γ secretion from CD8^+^ T cells	Skin tissue and blood from vitiligo patients	(55)
Cardiovascular inflammation	ISG15 form	ISG15 function	Research model	Refs
Inflammatory cardiomyopathy and heart failure	Conjugated	Induce a strong I-IFN response dependent on NF-κB	Mice myocarditis models induced by coxsackie B3 virus	(61)
		Suppress the release of infectious virus	Cardiomyopathy mice models	(60)
Viral cardiac inflammation	Conjugated	Inhibit glycolysis	Cardiovascular atrophy mice models induced by CVB-3 virus	(22)
Hypertension	Conjugated	Induce the production of vascular ROS and adverse vascular remodeling	Hypertension mice models; Angiotensin II (AngII)-treated vascular cells and macrophages.	(33, 64)
Non-compaction cardiomyopathy	Free	Decrease proliferation and increase maturation of cardiomyocyte	Transgenic embryonic mouse hearts.	(62)
Neuroinflammation	ISG15 form	ISG15 function	Research model	Refs
Aicardi-Goutières syndrome	N/A	The ISG15 levels increase in mice models	Aicardi-Goutières syndrome mice models	(65)
Demyelination	Conjugated and free	Activate microglia and alter miRNA composition in extracellular vesicles	Mouse models of demyelinating disease	(68)
Central nervous system infection	N/A	The ISG15 levels increase in infected rhesus macaques	Brain and blood cells of rhesus macaques infected by Simian immunodeficiency virus	(69)
Traumatic brain injury	N/A	The ISG15 levels increase in the cortex of traumatic brain injury mice	Injured cortex of aged mice caused by controlled cortical impact device.	(70)
Alzheimer disease	Conjugated	Inhibit autophagy	Brains of patients with Alzheimer disease; Alzheimer mice models	(31)
Experimental autoimmune encephalomyelitis	N/A	Co-localization with cyclic GMP-AMP synthase	Experimental autoimmune encephalomyelitis (EAE) mouse models	(76)
Other inflammatory diseases	ISG15 form	ISG15 function	Research model	Refs
Inflammatory bowel diseases	N/A	The ISG15 levels increase	Inflammatory bowel diseases	(36)
	N/A	The ISG15 levels increase in rat colon	Rat models of inflammatory bowel diseases exposed to Zearalenone	(77)
Rheumatoid arthritis and periodontal disease	N/A	The ISG15 levels increase	Blood samples of patients with rheumatoid arthritis and periodontal disease	(78)
Osteoarthritis	Conjugated	Promote cartilage degradation	Cartilage of osteoarthritis patients and rat models	(79)
Embryo-relevant inflammatory cytokine storms	Conjugated	Cause cytokine storm in the fetus	Beclin 1 deficient mouse embryos	(80)
Pancreatic cancer	Conjugated	Maintain the metabolic plasticity of pancreatic cancer stem cell	Blood samples of pancreatic cancer patients and mouse model	(83)

a. N/A, form of ISG15 not speciﬁed in the cited literature.

In the future, ISG15-related studies could focus on the dynamic regulatory mechanisms involved in noninfectious chronic inflammation, especially its interactions with cytokine networks and immune cell functions. Whether ISG15 can be used as a diagnostic biomarker and therapeutic intervention target for inflammatory diseases are the open questions remaining to assess and deserve more exploration. Cutting-edge technologies such as single-cell omics and spatial transcriptomics could be used to analyze the expression pattern and regulatory function of ISG15 in different cell types and inflammatory stages. Researches in this area will hopefully provide new theoretical basis and strategic support for precise treatment of clinical inflammatory diseases.
